# Mechanistic blockade of *Pseudomonas aeruginosa* type III secretion by a monoclonal antibody targeting the pore size-determining domain of PcrV

**DOI:** 10.1128/aac.00405-25

**Published:** 2025-08-18

**Authors:** Yu Zhang, Shiyu Guo, Liwen Jiang, Siqi Wang, Weitong Hou, Xiran Qiu, Hui Shen, Maomao An

**Affiliations:** 1 Department of Pharmacology, Shanghai Tenth People’s Hospital, Tongji University School of Medicine 481875, Shanghai, People’s Republic of China; 2 Department of Pharmacy, Ruijin Hospital, Shanghai Jiao Tong University School of Medicine 56694, Shanghai, People’s Republic of China; 3 Starmab Biologics (Suzhou) Co., Ltd., Shanghai, China; 4 Department of Clinical Laboratory Medicine, Shanghai Tenth People’s Hospital, Tongji University School of Medicine 481875, Shanghai, People’s Republic of China; Providence Portland Medical Center, Portland, Oregon, USA

**Keywords:** *Pseudomonas aeruginosa*, type III secretory system, PcrV, monoclonal antibody, translocon

## Abstract

*Pseudomonas aeruginosa* bloodstream infections carry mortality rates exceeding 60%, with escalating antibiotic resistance dramatically limiting therapeutic options. The type III secretion system (T3SS), a virulence apparatus delivering cytotoxic effectors via PcrV-dependent translocation pores, represents a therapeutic target. Here, we developed a monoclonal antibody (5C8) targeting the central domain (H106-D173) of PcrV, which regulates translocation pore size. 5C8 demonstrated sub-nanomolar affinity (KD = 0.32 nM) via biolayer interferometry and broad neutralization efficacy against clinical isolates (IC_50_: 0.32–1.47 μg/mL). In murine bloodstream infection models, 5C8 conferred improved survival against cytotoxic (*exoU^+^*) and invasive (*exoS^+^*) strains (*P* < 0.01 vs controls), reducing bacterial loads in lungs/kidneys by 1.5-log_10_ colony-forming unit (*P* < 0.01) and suppressing interleukin-6 levels by 60–82% (*P* < 0.01). Mechanistic studies revealed 5C8’s dual action: blocking effector release (ExoU/ExoT reduced by 41–88% via liquid chromatography-mass spectrometry) and constricting T3SS pores below 1.2 nm (carbohydrate exclusion assay). Molecular docking identified D125/K129/Y145 as critical binding residues, validated by alanine scanning and mutant construction. Humanized Hu5C8 retained potency (KD = 0.55 nM) with extended half-life (t_1/2_ = 91.26 h) through Fc receptor engineering. As an inhibitor targeting the pore size-determining domain of PcrV, 5C8 disrupts virulence through a novel dual mechanism, providing a paradigm-shifting strategy against multidrug-resistant *P. aeruginosa*, bridging a critical gap in sepsis management.

## INTRODUCTION

Bloodstream infections (BSIs) constitute a formidable global health challenge, ranking among the top seven causes of mortality in North America and Europe (1). *Pseudomonas aeruginosa*, responsible for 3–4% of Gram-negative bacteremia cases, carries a particularly grave prognosis with mortality rates reaching 21–62% due to its formidable antibiotic resistance profile (2–4). The World Health Organization elevated the threat level of carbapenem-resistant *P. aeruginosa* in 2017, designating it as a critical priority pathogen on the inaugural priority pathogens list (5). This classification reflects the urgent need for innovative therapeutic strategies.

Central to *P. aeruginosa* pathogenesis is the type III secretion system (T3SS), a molecular syringe mediating direct toxin delivery into host cells. This virulence apparatus deploys four canonical effector proteins (exoenzyme U [ExoU], ExoS, ExoT, and ExoY) that subvert epithelial integrity and immune defenses by inducing pleiotropic cell death modalities, including both programmed (e.g., apoptosis) and lytic pathways (6–9). Clinical evidence establishes T3SS expression as an independent mortality predictor in bacteremia patients (10–12), with the mutually exclusive *exoU*/*exoS* genotypes suggesting evolutionary optimization of distinct virulence strategies (13). While *exoT* and *exoY* maintain universal presence, the *exoU*/*exoS* dichotomy correlates with differential clinical outcomes (13).

The T3SS can be functionally divided into five components: the needle complex, effector proteins, translocon, regulatory proteins, and chaperones (14). The translocon, comprising PopB, PopD, and PcrV, not only mediates effector protein translocation but also directly induces host cell death. PopB and PopD hetero-oligomerize to form a 2.2–6.0 nm transmembrane pore, while PcrV acts as an essential scaffold for pore assembly (15, 16). Structural predictions suggest that PcrV adopts a dumbbell-shaped conformation organized into four domains: N-terminal (M1-K127), Helix-7 (R128-A158), C-terminal (K159-P250), and Helix-12 (L251-I294) (17–19). The C-terminal domain interacts with ‌PopB‌, mediating the formation of the ‌translocation pore (16)‌, while the N-terminal domain binds to ‌PopD‌, triggering the expression and secretion of effectors (20). The Helix-12 mediates PcrV pentamerization (21), forming a ring-like anchor securing the translocon to the T3SS needle tip (22). Crucially, functional studies confirm that the central domain (H106-D173) regulates pore size in a dose-dependent manner, with larger pores enhancing both toxin flux and host cell lysis (23).

Current empiric regimens (e.g., cephalosporin-aminoglycoside combinations) face dual challenges: rising antimicrobial resistance and endotoxin-induced septic shock (24, 25). Although neutralizing antibodies show promise in sepsis management, extracellular toxin targeting proves ineffective against T3SS-mediated intracellular delivery. Collectively, these limitations support PcrV as a strategic therapeutic target—inhibiting translocon assembly could simultaneously block toxin injection and reduce bacterial cytotoxicity. In this study, we identified a monoclonal antibody (mAb) 5C8 targeting PcrV that effectively protected mice from *P. aeruginosa* (ExoU/ExoS-expressing strains) BSI. We also investigated the mechanisms by which 5C8 inhibited *P. aeruginosa* virulence, including carbohydrate exclusion assays and molecular docking technology.

## RESULTS

### Prevalence of T3SS genes in clinical isolates of *P. aeruginosa*

**Fig 1:**
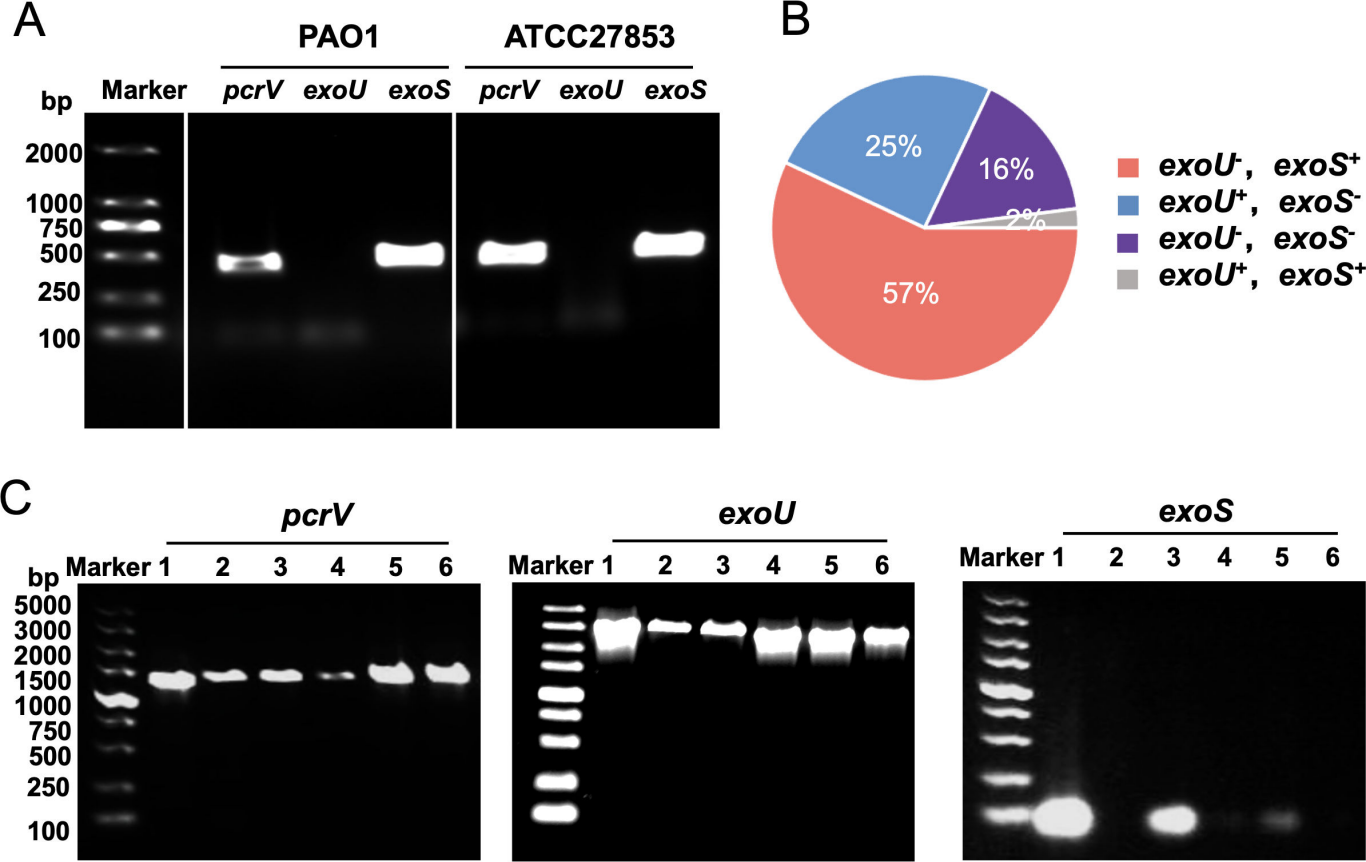
Detection of T3SS genes in *P. aeruginosa*. (**A**) PCR results for the *pcrV*, *exoU,* and *exoS* genes in standard strains. (**B**) Distribution of *exoU* and *exoS* genes among 147 clinical strains. (**C**) PCR results for the *pcrV*, *exoU,* and *exoS* genes in six clinical strains. Lanes 1 through 6 in each map represents strain 103753, 105275, 107979, 101553, 104375, and 105741, respectively.

To rigorously assess the inhibitory effects of anti-PcrV antibodies on T3SS, we conducted a comprehensive screen to identify cytotoxic (*exoU^+^*) strains for establishing a hemolysis model. Initially, we confirmed the genotypes of standard strains PAO1 and ATCC27853 as *pcrV*^+^/*exoS*^+^/*exoU*^-^ (Fig. 1A), aligning with previous studies (26, 27). Among 147 clinical isolates (sources: 119 sputum, 15 pus, 5 urine, 3 bile, 2 bronchoalveolar lavage fluid, and 1 each from ascites/wound/catheter) (Table S1), we prioritized *exoU*/*exoS* genotyping for cytotoxic strain selection. The gene distribution revealed interesting patterns: 2% were dual *exoS*^+^/*exoU*^+^ (*n* = 3), 16% lacked both (*n* = 24), 25% harbored only *exoU* (potential for hemolysis models) (*n* = 37), and 57% carried only *exoS* (*n* = 83) (Fig. 1B), supporting previous data and highlighting the heterogeneity within *P. aeruginosa* populations (13). Subsequent *pcrV* screening in 37 isolates identified as *exoU^+^*/*exoS^-^* revealed 37 (100%) carried *pcrV*, consistent with epidemiological reports (Table S1) (18, 26). PCR results for cytotoxic strains 103753, 105275, 107979, 101553, 104375, and 105741 are shown in Fig. 1C.

### Screening process of the 5C8 candidate

**Fig 2:**
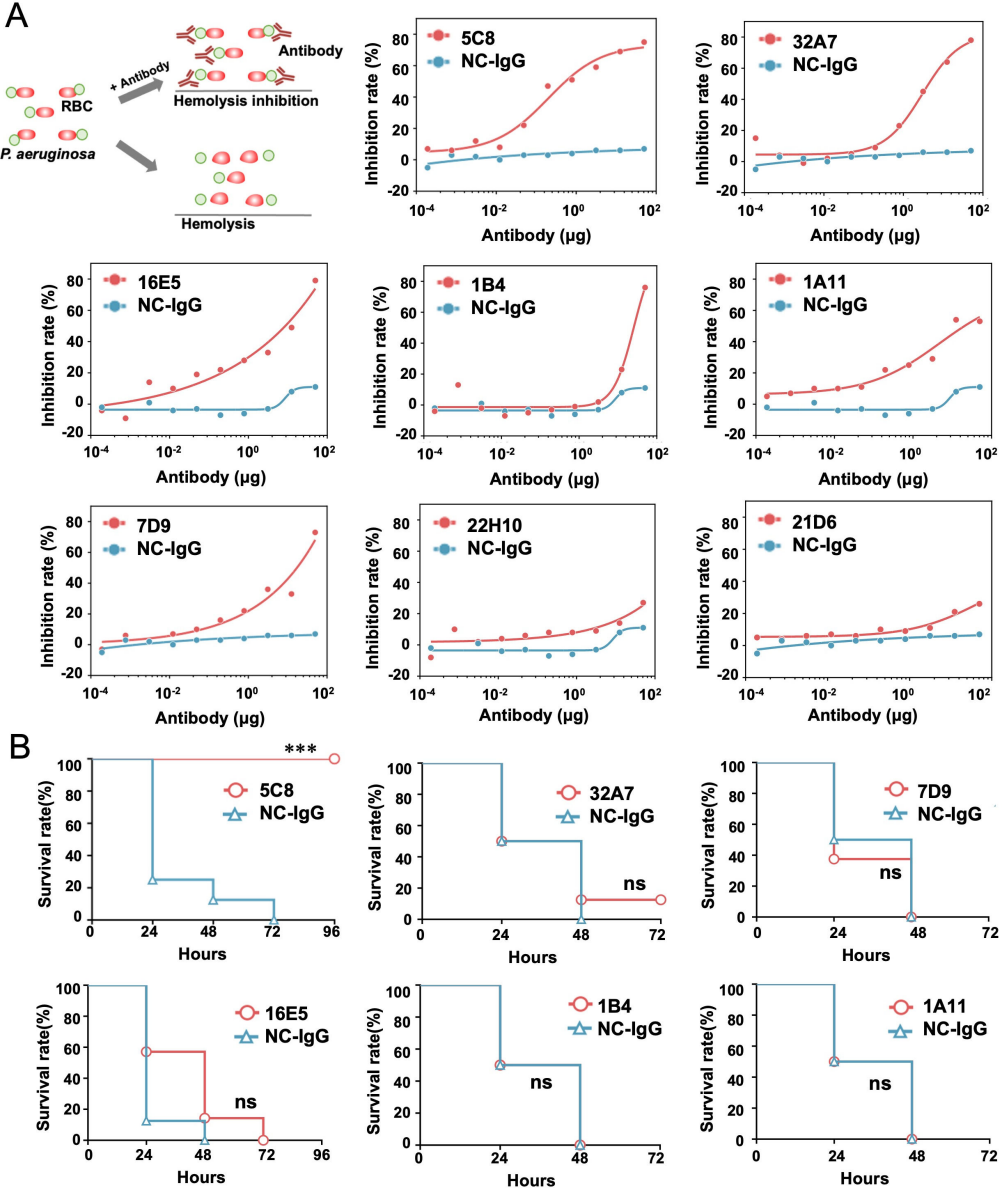
Evaluation of neutralizing activity and anti-infective efficacy of anti-PcrV mAbs against *P. aeruginosa*. (**A**) Illustration of the experiment and dose-response curves for neutralizing hemolytic effect of strain 103753 by anti-PcrV mAbs. The inhibition rate (%) was calculated by comparing the OD_405_ values of the test wells (containing anti-PcrV mAbs) to the OD_405_ values of the control wells (which received no IgG). (**B**) Survival curves of murine BSI model caused by strain 103753 (7.2 × 10^7^ CFU/mouse) and treated with anti-PcrV mAbs (25 mg/kg). ***P < 0.001, by the log-rank test. RBC, red blood cell; mAbs, monoclonal antibodies; NC-IgG, negative control immunoglobulin G.

To obtain candidate antibodies, we used hybridoma fusion technology, which enabled us to screen and prepare eight mAbs targeting PcrV. To establish their pharmacodynamic basis, we evaluated their affinity using enzyme-linked immunosorbent assay (ELISA), finding median effective concentration (EC_50_) values ranging from 5.732 to 39.56 ng/mL for PcrV binding (Table S2), indicating high affinity. Next, we tested these mAbs for inhibiting T3SS-mediated red blood cell (RBC) lysis by the *exoU^+^* strain 103753 and compared their inhibitory activities (Fig. 2A). Selected mAbs with *in vitro* T3SS inhibition were further assessed in a murine BSI model. Mice (*n* = 5) were inoculated with *P. aeruginosa* strain 103753 (7.2 × 10^7^ colony-forming unit [CFU]/mouse) and treated with mAbs at 25 mg/kg 1 h later (28). Survival differences within 4 days were analyzed using the log-rank test, revealing significant protection only with mAb 5C8 (*P* < 0.001) (Fig. 2B).

### 5C8 exhibits high affinity and potent *in vitro* neutralizing activity against *P. aeruginosa* isolates

**Fig 3:**
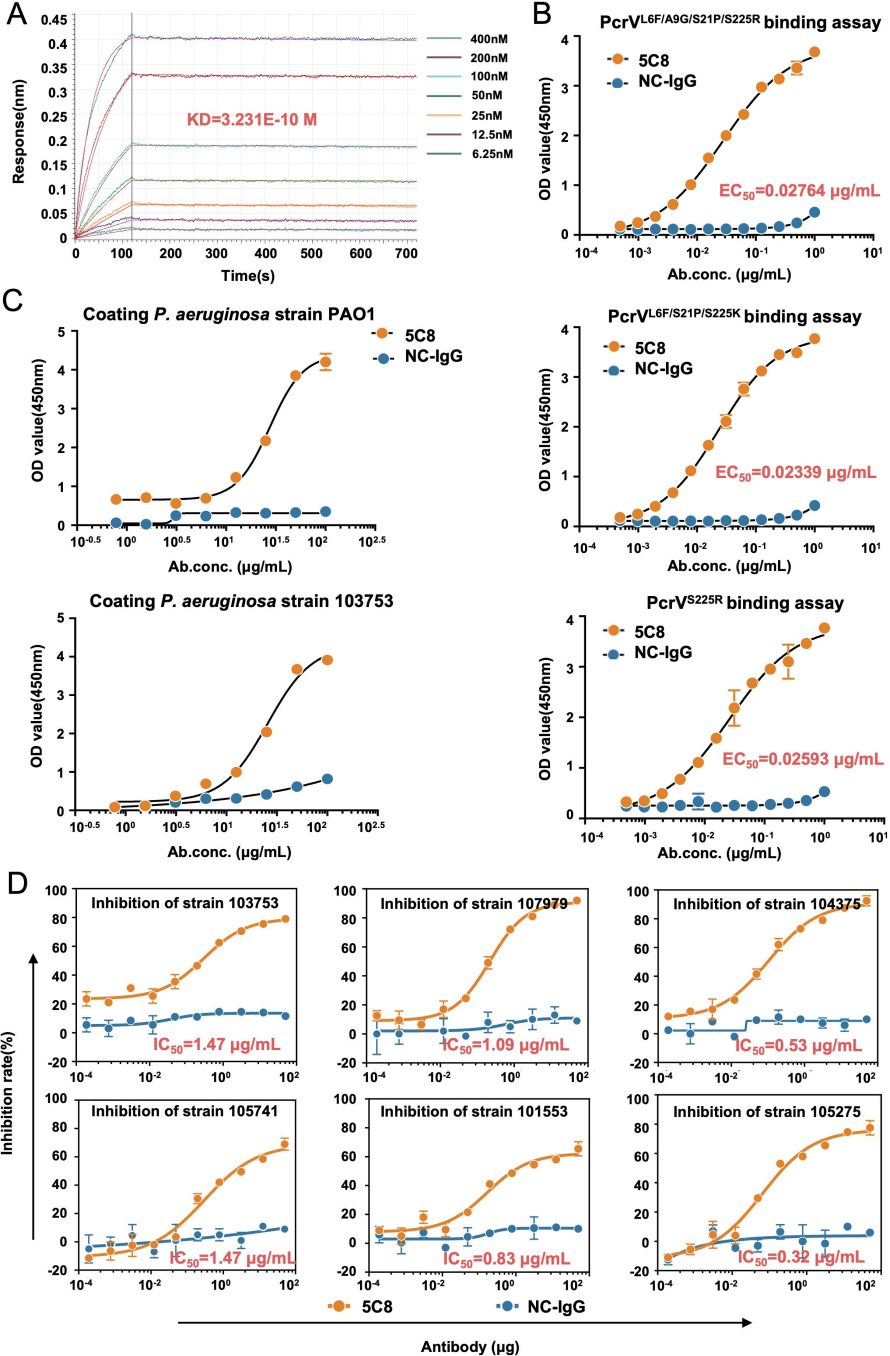
Evaluation of *in vitro* activity of 5C8. (**A**) Binding-dissociation kinetics curves of 5C8 and PcrV detected by BLI. (**B and C**) ELISA assays were conducted to confirm the binding of 5C8 to three PcrV mutants (**B**) and *P. aeruginosa* (**C**). (**D**) Dose-response curves of 5C8 for neutralizing the hemolytic effect of *P. aeruginosa* isolates. The inhibition rate (%) was calculated by comparing the OD_405_ values of the test wells (containing 5C8) to the OD_405_ values of the control wells (which received no IgG). The value in each map represents the average of two independent experiments. ELISA, enzyme-linked immunosorbent assay; NC-IgG, negative control immunoglobulin G.

Using biolayer interferometry (BLI), we monitored the real-time binding and dissociation of PcrV-5C8, revealing a high affinity (equilibrium dissociation constant [KD] = 0.3231 nM; Fig. 3A). To assess 5C8’s broad-spectrum activity against clinical *P. aeruginosa* isolates, we tested its affinity for three prevalent PcrV mutants (L6F/A9G/S21P/S225R, L6F/S21P/S225K, and S225R) with prevalences of 15.6%, 6.9%, and 6.5%, respectively (26, 29). Our results confirmed high affinity for all three mutants (Fig. 3B).

Additionally, 5C8 exhibited good binding activity towards *P. aeruginosa* strains carrying either the *exoS* or *exoU* gene (Fig. 3C) and strong specificity (Fig. S1), paving the way for its potential use in infection resistance. To assess clinical relevance, we tested 5C8’s neutralizing activity against six clinical *P. aeruginosa* isolates in RBC lysis inhibition assays. It protected RBCs from cytotoxicity with median inhibition concentration (IC_50_) values ranging from 0.32 to 1.47 µg/mL (Fig. 3D).

### 5C8 provides protection against both cytotoxic and invasive strains of *P. aeruginosa* in mice with BSIs

**Fig 4:**
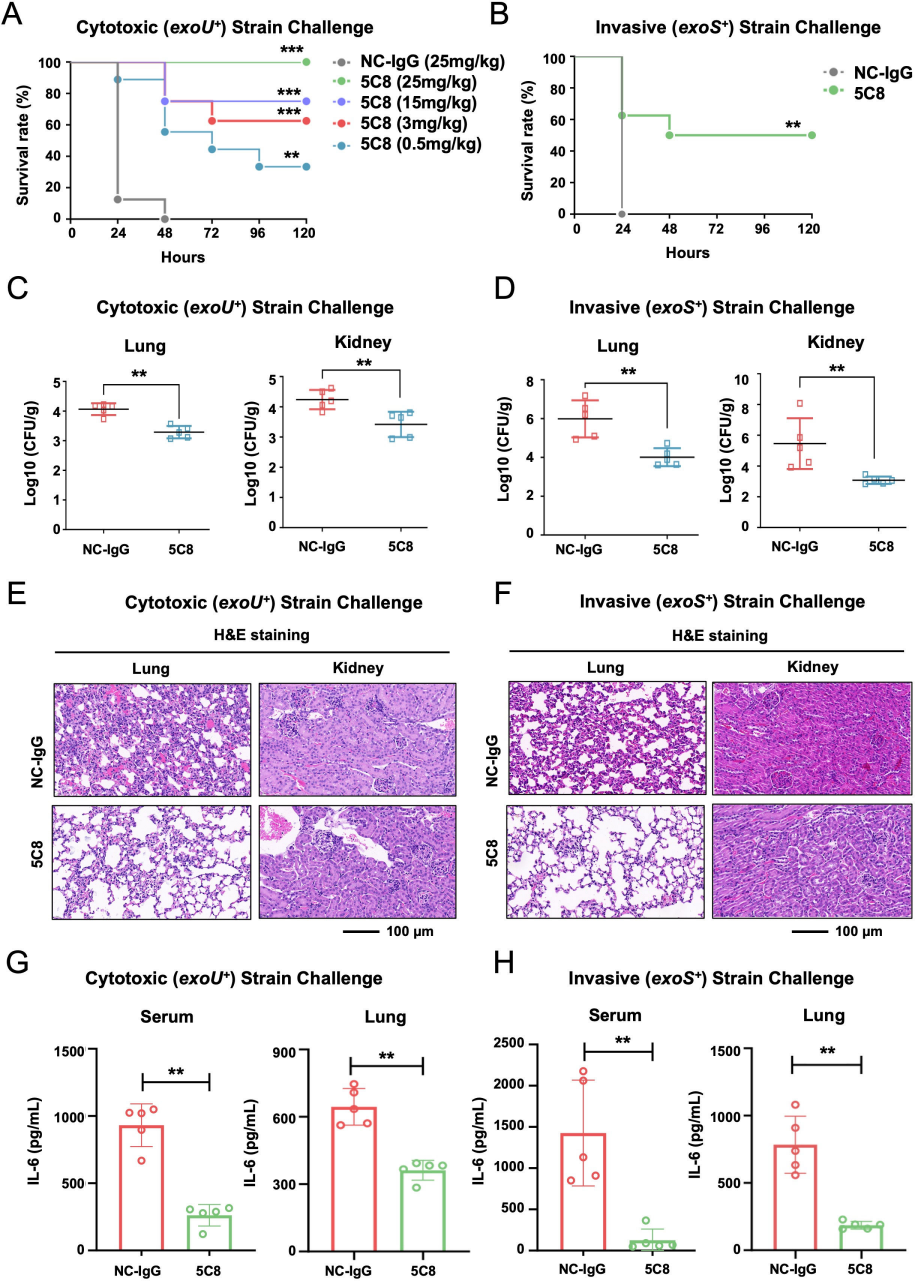
5C8 exhibits protective effects in murine BSI models. (**A**) Survival curves for mice (*n* = 8) infected with strain 103753 (7.2 × 10^7^ CFU/mouse) and treated with 5C8. **P < 0.01, ***P < 0.001; by the log-rank test. (**B**) Survival curves for mice (*n* = 8) infected with strain 100142 (6 × 10^7^ CFU/mouse) and treated with 5C8 at 25 mg/kg. **P < 0.01, by the log-rank test. (**C through H**) Mice (*n* = 5) were inoculated intravenously with either strain 103753 (3.6 × 10^7^ CFU/mouse) (**C, E, G**) or strain PAO1 (9.6 × 10^7^ CFU/mouse) (**D, F, H**), and treated with 5C8 at 25 mg/kg 1 h post-infection. Differences in bacterial burden in lung and kidney tissues of mice after 24 h of infection were calculated by the Mann-Whitney U test for 5C8 versus NC-IgG (**P < 0.01) (**C and D**). Representative hematoxylin and eosin (H&E) staining of lung and kidney tissues from mice after 24 h of infection (**E and F**). The IL-6 levels in serum and lung tissues from mice after 24 h of infection were quantified by ELISA. **P < 0.01, by the Mann-Whitney U test (**G and H**). NC-IgG, negative control immunoglobulin G.

The efficacy of 5C8 against *P. aeruginosa* was evaluated in BSI models induced by cytotoxic (*exoU^+^*) and invasive (*exoS^+^*) strains. In the cytotoxic strain-induced model, mice challenged with *P. aeruginosa* strain 103753 were monitored for survival after administering varying doses of 5C8 or NC-IgG. Compared to NC-IgG, 5C8 significantly protected mice at all doses (*P* = 0.0012 at 0.5 mg/kg, *P* = 0.0002 at 3 and 15 mg/kg, and *P* < 0.0001 at 25 mg/kg; Fig. 4A). At 25 mg/kg, 5C8 significantly reduced bacterial burden and pathological damage in the lungs and kidneys (*P* < 0.01; Fig. 4C and E), and decreased the systemic inflammatory response, as evidenced by lower cytokine levels in both plasma and lung homogenates (mean interleukin-6 [IL-6] concentration: 5C8 261.90/361.98 pg/mL vs NC-IgG 932.03/644.90 pg/mL, *P* < 0.01; Fig. 4G). In the invasive strain-induced model, mice challenged with *P. aeruginosa* and treated with 5C8 at 25 mg/kg had higher survival rates (*P* < 0.01; Fig. 4B), reduced bacterial burden and pathological damage in the lungs and kidneys (*P* < 0.01; Fig. 4D and F), and lower cytokine levels (mean IL-6 concentration: 5C8 126.99/185.65 pg/mL vs NC-IgG 1425.90/784.09 pg/mL, *P* < 0.01; Fig. 4H).

### The Fc domain does not contribute to the efficacy of 5C8 against *P. aeruginosa* infections

**Fig 5:**
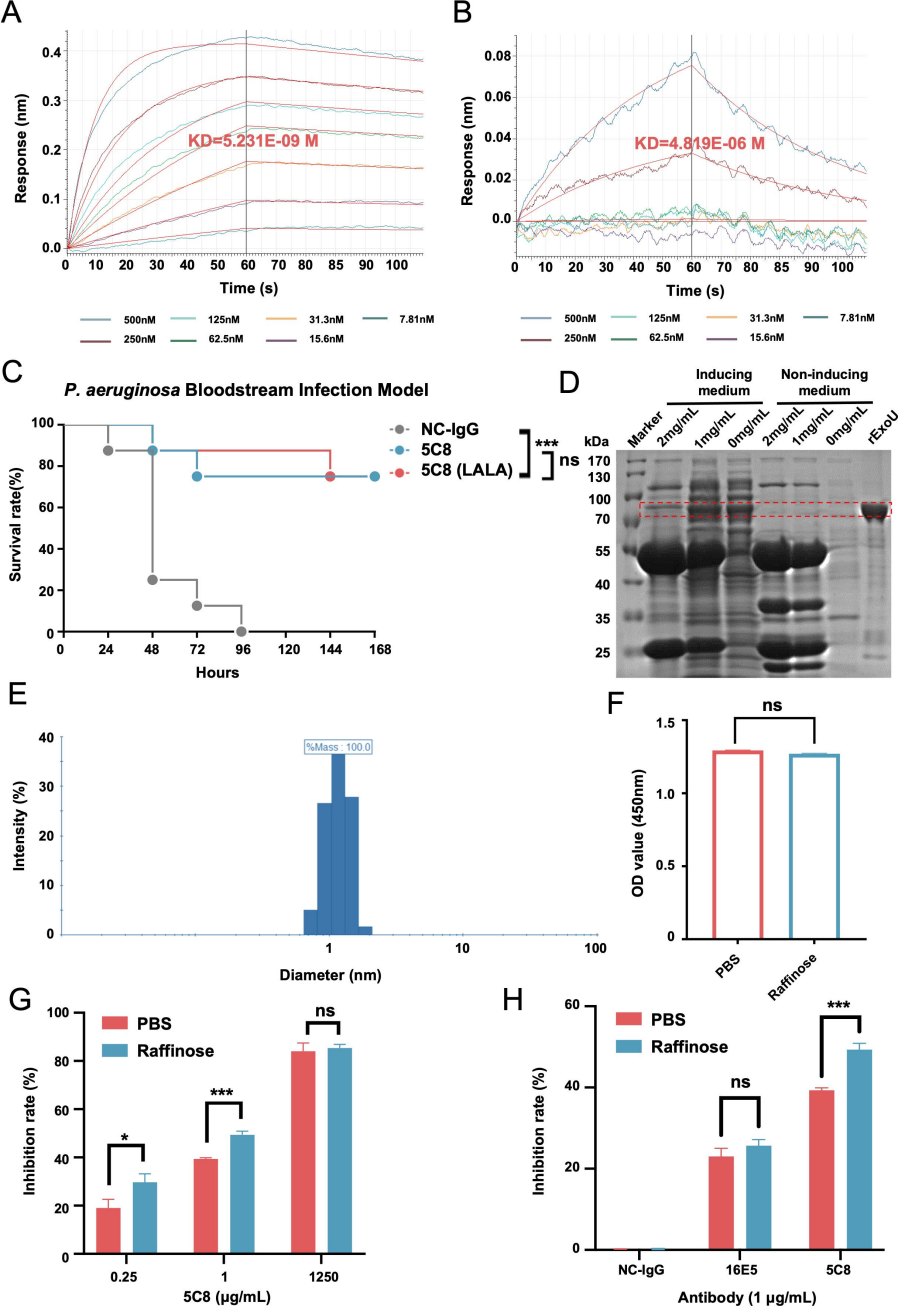
Mechanism of 5C8 blocking the function of T3SS. (**A and B**) The binding-dissociation kinetics curves of FcγRI (CD64) with 5C8 (**A**) and 5C8 (LALA) (**B**) detected using BLI. The KD value represents the average of two independent experiments. (**C**) Survival curves for mice (*n* = 8) infected with strain 103753 (7.2 × 10^7^ CFU/mouse) and treated with 5C8 or 5C8 (LALA) at 25 mg/kg. *P* > 0.05, ***P < 0.001; by the log-rank test. (**D**) Sodium dodecyl sulfate polyacrylamide gel electrophoresis analysis of ExoU release from *P. aeruginosa* strain 103753. Bacteria grown under T3SS-inducing or non-inducing conditions were exposed to 5C8 (0, 1, or 2 mg/mL). Recombinant ExoU, which has a theoretical monomer molecular weight of ~74 kDa, serves as a migration reference for native ExoU. (**E**) The hydrodynamic size of raffinose was determined by dynamic light scattering. (**F**) Hemolysis inhibition assay evaluating raffinose against *P. aeruginosa.* Phosphate buffered saline (PBS) served as the negative control. *P* > 0.05, by the two-tailed Student’s t-test. (**G and H**) Hemolysis inhibition assays. Activity of 5C8 (0.25, 1, and 1250 µg/mL) with or without raffinose (PBS) (**G**).Activity of anti-PcrV antibodies (16E5 and 5C8, 1 µg/mL) with or without raffinose (PBS), NC-IgG served as control (**H**). *P* > 0.05, *P < 0.05, and ***P < 0.001; by the two-tailed Student’s t-test. Data are presented as mean ± SD. NC-IgG, negative control immunoglobulin G.

Previous research has shown that mutating residues 234 and 235 of the crystallizable fragment (Fc) to alanine (LALA mutation) disables antibody binding to Fcγ receptors (FcγRs), eliminating the antibody-dependent enhancement (ADE) effect, while preserving binding to the neonatal Fc receptor (FcRn) (30–33). To determine whether the Fc domain of 5C8 is crucial for its anti-infective effects, we created 5C8 (LALA), which lost FcγR binding capacity. As shown in Fig. 5A and B, wild-type 5C8 exhibited strong binding to FcγRI (KD = 5.231E-09 M), whereas 5C8 (LALA) showed weak binding (KD = 4.819E-06 M). Notably, in a murine BSI model, treatment with 5C8 (LALA) significantly improved survival rates, with no significant difference compared to 5C8 (*P* = 0.9688; Fig. 5C). These experiments demonstrate that 5C8’s efficacy in inhibiting T3SS-mediated anti-*P. aeruginosa* infection does not depend on its Fc domain binding to FcγRs to mediate antibody-dependent cell-mediated cytotoxicity, antibody-dependent cell-mediated phagocytosis, or related functions.

### 5C8 exerts dual inhibitory effects on T3SS by blocking ExoU release and constricting translocation pore dimensions

Mechanistically, PcrV coordinates two critical T3SS functions: (i) effector secretion triggering by interaction of PopD-PcrV (20)‌, and (ii) pore assembly via PopB/PopD oligomerization (16). To dissect 5C8’s mode of action, we systematically evaluated its impact on these two processes.

To assess 5C8’s interference with T3SS-dependent effector secretion, we quantified secreted proteins in bacterial supernatants. Sodium dodecyl sulfate polyacrylamide gel electrophoresis (SDS-PAGE) showed decreasing secreted ExoU levels under T3SS-inducing conditions with higher 5C8 concentrations, whereas ExoU was undetectable under non-inducing conditions (Fig. 5D). Liquid chromatography-mass spectrometry (LC-MS) further validated the depletion of key effectors: ExoU (41% reduction), ExoT (88% reduction), and ExoY (below detection limit) compared to untreated controls (Table 1). This demonstrates that 5C8 directly disrupts effector flux independent of pore activity.

**TABLE 1:** Identification of T3SS effectors in the culture supernatant of *P. aeruginosa* by LC-MS^*b*^

Function	Accession	Protein	MW (kDa)	Abundance		
				PA	PA + 5C8	(PA + 5C8)/PA
Adhesion factor	A0A060GS31	Flagellin	39.4	1.06E10	1.17E10	110%
T3SS effector protein	O34208	ExoU	73.9	7.56E11	4.46E11	59%
	Q9I1S4	ExoY	41.7	1.06E8	-^*a*^	
	Q9I788	ExoT	48.5	7.63E11	8.88E10	12%

^*a*^ The “-” indicates the abundance dropped below the detection limit.

^*b*^ T3SS, type III secretion system; PA, *Pseudomonas aeruginosa*.

To isolate 5C8’s effect on pore dimensions, we used a carbohydrate exclusion assay (a commonly employed method for studying pore size) (23, 34, 35). Raffinose, measured to be approximately 1.2 nm in diameter (smaller than the T3SS pore size in host cell membranes), was confirmed not to physically block the pore due to its diameter being less than 2.2 nm (Fig. 5E and F). Strikingly, subinhibitory concentrations of 5C8 (0.25 or 1 µg/mL) were synergized with raffinose to enhance RBC protection (Fig. 5G; *P* < 0.01 vs 5C8 alone). This synergistic inhibition was specific to 5C8, as no comparable effect was observed with anti-PcrV antibody (16E5) (Fig. 5H). This indicates that 5C8 physically constricts pores to diameters below 1.2 nm—a size insufficient for effector translocation (given typical T3SS pore diameters > 2.2 nm)—thereby independently preventing pore-mediated cytotoxicity.

Collectively, these orthogonal approaches demonstrate 5C8’s dual inhibition: (i) effector secretion blockade: 5C8 reduces effector release by >40% (ExoU) through PcrV-targeted interference with secretory machinery, and (ii) pore size restriction: 5C8 sterically limits pore diameter below 1.2 nm, physically blocking residual effectors from traversing the host membrane.

### 5C8 specifically targets key residues within the translocation pore size-determining domain of PcrV

**Fig 6:**
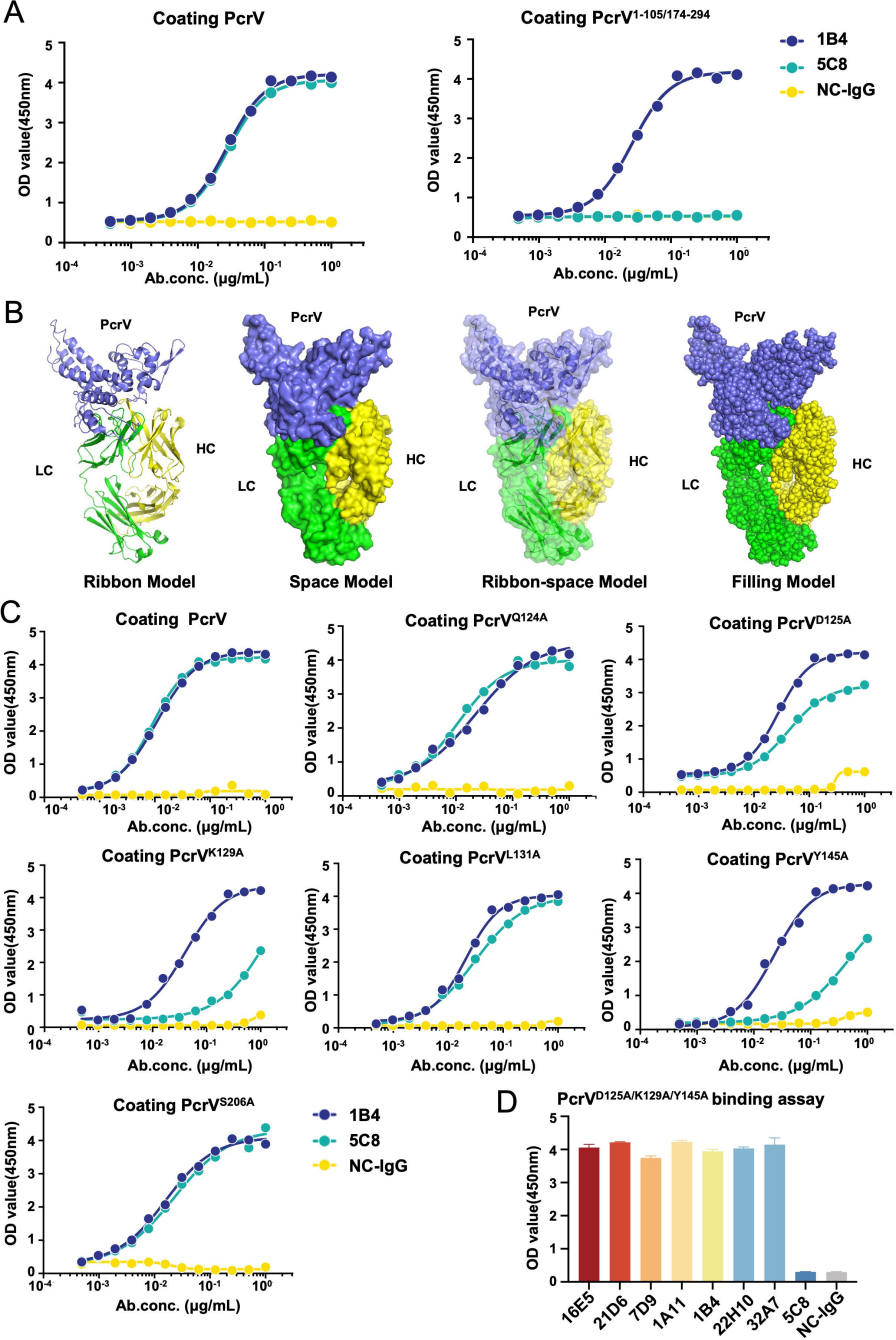
Determination of 5C8-binding epitopes. (**A**) ELISA was used to verify the affinity of 5C8 for PcrV^1-105/174-294^. (**B**) Three-dimensional structure of 5C8-Fab complexed to PcrV. The light chain (LC) is green, the heavy chain (HC) is yellow, and PcrV is purple. (**C**) ELISA assays were conducted to confirm the binding of 5C8 to PcrV mutants. (**D**) ELISA was performed with anti-PcrV mAbs (1 µg/mL) and PcrV^D125A/K129A/Y145A^. Wild-type PcrV and mAbs (16E5, 21D6, 7D9, 1A11, 1B4, 22H10, and 32A7) served as positive controls. Data are presented as mean ± SD. ELISA, enzyme-linked immunosorbent assay; mAbs, monoclonal antibodies; NC-IgG, negative control immunoglobulin G.

Based on the aforementioned findings, we hypothesized that 5C8 specifically binds to the translocation pore size-determining domain (H106-D173) of PcrV. To validate this, we first constructed a deletion mutant of this domain (PcrV^1-105/174-294^) and assessed antibody binding via ELISA. The results demonstrated that the positive control mAb 1B4 retained strong binding affinity for the truncated PcrV, whereas 5C8 exhibited no detectable binding activity (Fig. 6A), confirming that 5C8 binding is strictly dependent on the H106-D173 domain. To further identify critical residues mediating this interaction, we performed template-based structural modeling of PcrV and 5C8, followed by molecular docking analysis. The predicted complex structure (Fig. 6B) highlighted six potential binding residues in PcrV (Q124, D125, K129, L131, Y145, and S206). Among these, DS software-based energy calculations prioritized D125, K129, and Y145 as key contributors to the interaction (Table S3). To experimentally validate these predictions, we generated alanine substitution mutants of all six residues in full-length PcrV and tested their binding to 5C8 using ELISA. Strikingly, mutations at D125, K129, or Y145 (PcrV^D125A^, PcrV^K129A^, and PcrV^Y145A^) abolished 5C8 binding while retaining strong binding to 1B4. In contrast, mutations at Q124, L131, or S206 (PcrV^Q124A^, PcrV^L131A^, PcrV^S206A^) showed no impairment of binding to either antibody (Fig. 6C). Energy perturbation analysis further revealed that substitutions at D125, K129, or Y145 with non-alanine residues maximally disrupted interaction energies, eliminating all stabilizing contacts between PcrV and 5C8 (Fig. S2). Critically, combinatorial mutation of all three residues (D125, K129, and Y145) completely abrogated 5C8 binding (Fig. 6D), consistent with the behavior of negative control IgG (NC-IgG). Protein expression and purity of all mutants were verified by SDS-PAGE (Fig. S3). Collectively, these structural and functional analyses unequivocally identify D125, K129, and Y145 within the H106-D173 domain as essential residues governing the specific interaction between PcrV and 5C8.

### Characterization of Hu5C8

**Fig 7:**
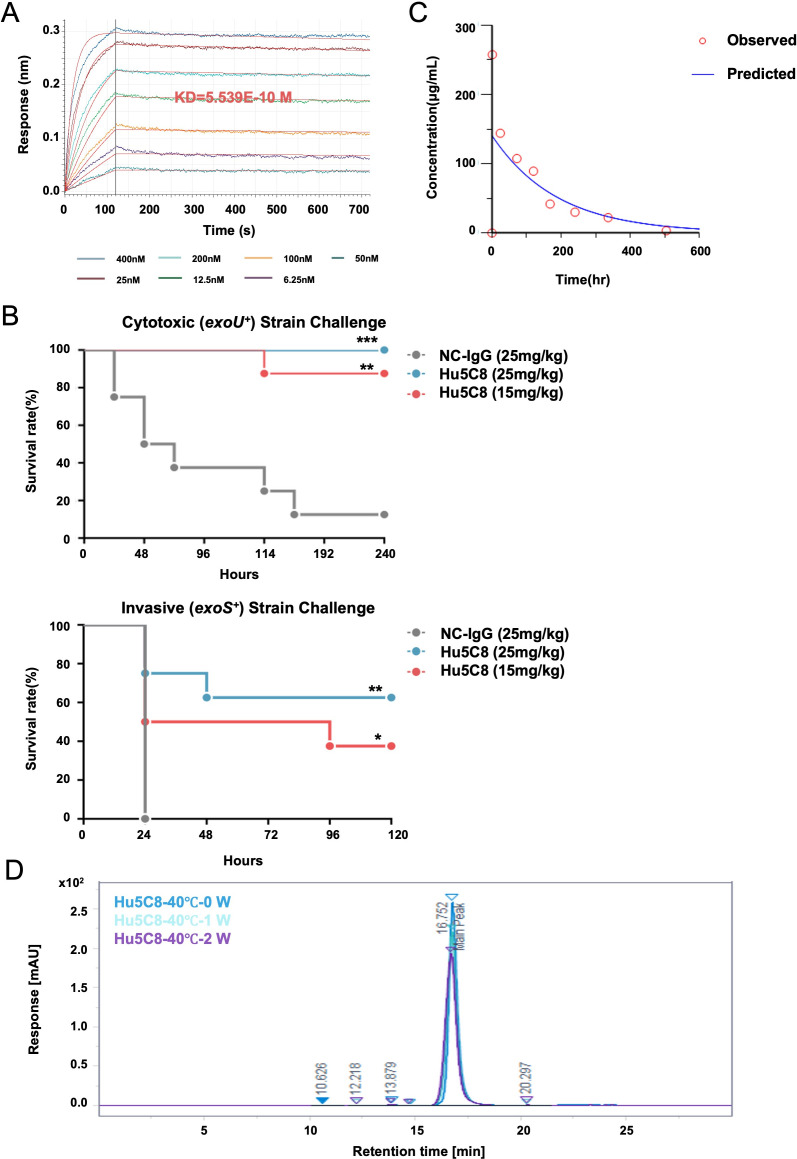
Characterization of Hu5C8. (**A**) The binding-dissociation kinetics curves of PcrV and Hu5C8 detected by BLI. (**B**) Survival curves for mice with cytotoxic (upper) and invasive (lower) *P. aeruginosa* strain treated with Hu5C8. Mice (*n* = 8) were inoculated intravenously with strain 103753 (7.2e7 CFU) or 100142 (6e7 CFU), and treated with Hu5C8 at 25 and15 mg/kg for 1 h post-infection. *P < 0.05, **P < 0.01, ***P < 0.001; by the log-rank test. (**C**) Mean serum concentration-time curve (fitting) of Hu5C8 in C57BL/6N mice (*n* = 3) following a single intravenous dose of 15 mg/kg. Serum concentrations were quantified by ELISA and modeled using Phoenix 8.1. Data are presented as mean ± SD. (**D**) Overlay of SEC-HPLC chromatograms for Hu5C8 samples stored at 40°C for two weeks. ELISA, enzyme-linked immunosorbent assay; SEC-HPLC, size exclusion high-performance liquid chromatography.

Building upon the murine 5C8 framework, we successfully engineered Hu5C8—a humanized variant demonstrating superior developability profiles. Biophysical characterization revealed that Hu5C8 maintained sub-nanomolar affinity (KD = 0.5439 nM; Fig. 7A) while exhibiting <1% high-molecular-weight species after accelerated stability testing (40°C/75% RH for 2 weeks; Fig. 7D). Notably, Hu5C8 neutralized both *exoU^+^* and *exoS^+^* clinical isolates with comparable efficacy (Fig. 7B), suggesting its T3SS-centric mechanism bypasses strain-specific effector protein variations—a distinct advantage over therapies targeting individual virulence factors. Pharmacokinetic studies in mice revealed Hu5C8’s extended half-life (t_1/2_ = 91.26 h, Fig. 7C) through FcRn-engineering (YTE mutation) (36), potentially enabling single-dose regimens for BSIs.

## DISCUSSION

The emergence of multidrug-resistant *P. aeruginosa* BSIs demands innovative therapeutic strategies that circumvent traditional antibiotic resistance mechanisms (37). Our study presents 5C8 as a novel mAb targeting the pore size-determining domain (H106-D173) of PcrV, demonstrating a dual mechanism of pore size restriction and effector secretion blockade. This finding expands the therapeutic potential beyond existing PcrV-targeting agents like KB001-A (a modified fragment of mAb166) and MEDI3902, particularly in acute infection settings where T3SS plays a pivotal role.

Although the binding regions of the previous PcrV-targeted antibodies overlap with the pore size-determining domain (H106-D173) (e.g., mAb166 binds to residues 144-257) (17, 18, 23, 26, 38, 39), the specific binding of 5C8 to residues D125, K129, and Y145—completely localized within this subdomain (Fig. 6)—introduces a unique therapeutic paradigm. This dual mechanism of pore constriction (to <1.2 nm; Fig. 5E through H) and effector secretion blockade (Fig. 5D; Table 1) has not been demonstrated for previous anti-PcrV antibodies. Importantly, this dual-blocking activity is Fc-independent (Fig. 5A through C), distinguishing 5C8 from MEDI3902, a bispecific antibody that relies on opsonic Fc-mediated effector functions (40). By decoupling neutralization from Fc-dependent mechanisms, 5C8 mitigates potential risks of ADE observed in some Fc-dependent therapies (31, 33), a critical safety advantage for clinical applications.

Our murine models demonstrated 5C8’s superiority over KB001-A (100% vs 87.5%) in improving survival, though its efficacy remained modest compared to MEDI3902 (Fig. S4). This discrepancy likely stems from MEDI3902’s dual targeting of PcrV and Psl exopolysaccharide (26, 27), highlighting opportunities for 5C8-based combination therapies. Notably, while MEDI3902’s phase II trial challenges were multifactorial (including patient selection and endpoint design) (41), 5C8’s specific targeting of acute-phase virulence factors makes it particularly suited for bacteremia management—a population excluded from prior T3SS-targeting trials (38).

Three key findings support clinical development: (i) Broad strain coverage demonstrated by 5C8’s maintained binding to prevalent PcrV mutants (L6F/A9G/S21P/S225R) (Fig. 3B); (ii) cytokine modulation via 60–82% IL-6 reduction (*P* < 0.01) (Fig. 4G and H) suggests 5C8 may mitigate sepsis-associated cytokine storms—a critical advantage over pure bactericidal agents (42); and (iii) pharmacodynamic resilience: LALA-mutated 5C8 retained full efficacy (Fig. 5C), enabling Fc engineering to extend half-life without compromising function (31).

While our carbohydrate exclusion assay and molecular modeling support the pore size hypothesis (Fig. 5 and 6), cryo-EM studies are needed to visualize 5C8-PcrV interactions at atomic resolution. Additionally, combination studies with antipseudomonal β-lactams could exploit 5C8’s immunomodulatory effects to enhance bacterial clearance (43). Given the high mortality of carbapenem-resistant *P. aeruginosa* bacteremia (44), 5C8 warrants evaluation in neutropenic and post-transplant models where T3SS is predominant (45, 46).

## MATERIALS AND METHODS

### Bacterial strains and culture

The standard strains PAO1 and ATCC27853 of *P. aeruginosa* were provided by Professor Vasil ML (University of Colorado, Denver, USA) and the Clinical Laboratory Medicine of the Tenth People’s Hospital of Shanghai, China, respectively. Clinical isolation strains of *P. aeruginosa* were obtained from the latter institution. These strains were propagated on sheep blood agar plates or cultured in Luria-Bertani (LB) broth at 37°C. For hemolysis assays and infection model experiments, bacteria were grown under T3SS-inducing conditions in tryptic soy broth supplemented with 100 mM glutamine, 1% glycerol, and 10 mM nitrilotriacetic acid at 32°C (47).

### Genotyping

The presence of the *pcrV*, *exoU*, and *exoS* genes in all *P. aeruginosa* strains was determined through PCR, utilizing primer pairs as shown in Table S4. Bacteria were grown overnight at 37°C, and DNA was isolated by high temperature boiling of bacterial clones. The PCR was set up as follows: 1 µL DNA template (100 ng), 1 µL total PCR primers (a final 0.2 µM concentration of each primer), 25 µL Premix Taq Hot Start Version (Takara), and 23 µL sterile water. PCR amplification was carried out as follows: initial denaturation at 94°C for 5 min; 30 cycles of 94°C for 30 s, 55°C for 30 s, and 72°C for 1 min/1000 bp; and a final extension step at 72°C for 7 min. Strains that were *pcrv^+^*/*exoU^+^*/*exoS^-^* were considered cytotoxic. *Pcrv^+^*/*exoS^+^*/*exoU^-^* strains were classified as invasive.

### Expression of recombinant PcrV

The sequence of the *pcrV* gene, retrieved from UniProt, was subsequently synthesized and cloned into the pET21a vector upstream of the 6xHis tag. This construct was transformed into *Escherichia coli* BL21(DE3) cells and expression induced by overnight culture in LB medium supplemented with 0.1 mM IPTG. Subsequently, the harvested cells were lysed using an ultrasonic cell crusher, and the soluble recombinant PcrV was purified via a Ni-NTA agarose column (Qiagen).

Recombinant mutants of PcrV^1-105/174-294^, PcrV^Q124A^, PcrV^D125A^, PcrV^K129A^, PcrV^L131A^, PcrV^Y145A^, PcrV^S206A^, and PcrV^D125A/K129A/Y145A^ were expressed using the identical methods as described above.

### Generation of mAbs

The recombinant PcrV protein was used to immunize BALB/c mice. After euthanasia, splenocytes isolated from these mice were fused with murine myeloma P3 × 63Ag8.653 cells to create hybridoma cells. RNA_fast200_ (Fastagen) was used to generate total RNA from the hybridoma cells, which was subsequently transcribed into cDNA serving as the template DNA in PCR reactions utilizing primer pairs previously described by Chardès et al. (48–50). The resulting amplified antibody sequences underwent gene synthesis and were fused with the constant regions of human IgG1 (immunoglobulin G 1), followed by their insertion into the pcDNA3.4 vector. Subsequently, these constructs were transiently transfected into Expi293F cells (Thermo Fisher) and cultured for 6 days. The supernatant containing the expressed antibodies was then purified using protein A magnetic beads (GenScript).

### Enzyme-linked immunosorbent assay

Recombinant wild-type PcrV or mutated PcrVs were coated in 96-well plates at a concentration of 0.1 µg/well and incubated overnight at 4°C. Subsequently, the plates were blocked with PBST (phosphate buffered saline [PBS] containing 0.05% Tween-20) supplemented with 5% BSA. Following this, the plates were incubated with a series of dilutions of mAbs for 1 h. Subsequently, horseradish peroxidase-labeled goat anti-human IgG (Thermo Fisher) was added and incubated for 1 h. Between each step, the plates were thoroughly washed five times with PBST. For detection, the plates were developed using TMB (3,3′,5,5′-tetramethylbenzidine) solution, and the absorbance was measured at 450 nm.

To determine the binding activity of 5C8 to *P. aeruginosa*, 96-well plates were coated with *P. aeruginosa* strains (1 × 10^9^ CFU/mL) using the protocol described previously. *Staphylococcus aureus* (USA300) and *E. coli* BL21(DE3) (both lacking *pcrV*) were included as negative controls to validate specificity.

### Hemolysis inhibition assay

The *P. aeruginosa* isolates were grown under inducing conditions overnight, then harvested by centrifugation and resuspended in PBS to reach a concentration of 2 × 10^8^ CFU/ml. In a 96-well plate, 50 µL of the bacterial suspension was mixed with 50 µL of diluted purified mAbs. Rabbit RBCs, washed with PBS and resuspended to 5% (vol/vol), were added at 100 µL per well containing the bacteria-antibody mixture. After thorough agitation and incubation at 37°C for 1.5 h, the plates were centrifuged. The supernatant was then transferred to a new plate, and its optical density at 405 nm (OD_405_) was measured using a plate reader.

### BLI assay

The binding affinity between PcrV and 5C8 was measured by BLI assay using the Octet RED384 system (Sartorius). In brief, the FAB2G biosensor was loaded with 5C8 (5 µg/mL). Subsequently, various concentrations of the PcrV protein, ranging from 6.25 nM to 400 nM, were added for 120 s, followed by a dissociation phase lasting 600 s. The captured data were then analyzed using the Octet Analysis Studio software to determine the KD values.

### Murine infection models

The murine infection models were induced using the cytotoxic (*exoU^+^*) strain 103753 of *P. aeruginosa*, as well as the invasive (*exoS^+^*) strains PAO1 and 100142. Bacteria were grown under inducing conditions overnight, then resuspended in PBS to specified density. Immunocompetent, 8-week-old, female C57BL/6 mice were infected intravenously (i.v.) via the lateral tail vein with *P. aeruginosa* suspended in a 200 µL inoculum of varying concentrations: either 7.2 × 10^7^ or 3.6 × 10^7^ CFU/mouse for strain 103753, 9.6 × 10^7^ CFU/mouse for strain PAO1, and 6 × 10^7^ CFU/mouse for strain 100142. mAbs were administered i.v. 1 h after infection. For the organ burden and histopathology experiments, the lungs and kidneys were harvested 24 h after infection for the determination of CFUs and staining with hematoxylin and eosin. For the quantification of IL-6, blood and kidneys were harvested 24 h after infection, and ELISA kits (Thermo Fisher) were used according to the manufacturer’s instructions.

### Analysis of T3SS effectors

*P. aeruginosa* strain 103753 was grown overnight under T3SS-inducing conditions or non-inducing conditions (as described above), with varying concentrations of 5C8 (0, 1, or 2 mg/mL added upon inoculation) (19). The supernatant was analyzed using SDS-PAGE and LC-MS. For SDS-PAGE, 20 µL of a 10-fold concentrated supernatant sample was loaded per lane of a 10% SDS-polyacrylamide gel and stained with Coomassie blue. The recombinant ExoU, prepared using the protocol described previously, served as a migration reference to confirm the position of native ExoU. To further quantify ExoU expression, the supernatant was digested with a protease for LC-MS analysis. Protein species identification and functional classification were completed using data from the UniProt database.

### Carbohydrate exclusion assay

Washed RBCs were resuspended in PBS alone or PBS with 60 mM raffinose to a final concentration of 5%. Then, 100 µL of the RBC suspension was mixed with 50 µL of *P. aeruginosa* strain 103753 (2 × 10^8^ CFU/mL) in individual wells of a 96-well plate (23, 34, 35). To this, 50 µL of anti-PcrV antibodies at varying concentrations (0.25, 1, or 1250 µg/mL) was added, agitated for homogeneity, and incubated at 37°C for 1.5 h. After centrifugation, 100 µL of supernatant from each well was transferred to a new plate and OD_405_ was measured.

### Homology modeling and molecular docking

The structural models for PcrV and 5C8 were built using BIOVIA Discovery Studio 2019 (DS). Template search and alignment were conducted within the DS template library, and highly homologous PDB structures were downloaded from RCSB (https://www.rcsb.org/). The models were constructed based on targeted-template alignment and validated using Ramachandran plots.

Molecular docking techniques can predict the binding mechanisms and activity of antibody-antigen interactions. To predict the interaction between 5C8 and PcrV, we used ZDOCK, a rigid docking tool. RDOCK, an energy optimization process based on CHARMm, was then employed to refine and score these binding configurations, which were saved as PDB files. Finally, DS was used to analyze the antibody-antigen contact regions between 5C8 and PcrV.

### Humanization of 5C8

The humanization of 5C8 was performed using a framework homology-based complementarity-determining region grafting approach with back-mutation optimization. Human IgG1 framework regions (FRs) with high sequence homology were selected via the IMGT/DomainGapAlign tool. Murine FRs were replaced with human IgG1 FRs while retaining critical murine residues for antigen-binding integrity, followed by fusion to human IgG1 constant regions (containing “YTE” mutation (36)). Following this, humanized antibody expression plasmids were constructed, and recombinant antibodies were generated in Expi293F cells. These antibodies were screened using SDS-PAGE for purity and ELISA for binding affinity, facilitating a rational selection process.

### Accelerated stability testing

Hu5C8 was diluted to 1 mg/mL using ultrapure water. Aliquots (100 µL each) were incubated in a temperature- and humidity-controlled chamber (40°C/75% RH) for 0, 1, and 2 weeks. Stability under accelerated stress conditions was evaluated by size exclusion high-performance liquid chromatography (Agilent) using a Waters SEC-200Å column (7.8 × 300 mm). Samples collected at each timepoint were analyzed against the time-zero control, with main peak retention time and monomer content (% area) serving as critical quality attributes.

### Determination of pharmacokinetic

C57BL/6N mice (*n* = 3) were administered with 15 mg/kg of Hu5C8 i.v. to assess its pharmacokinetic profile. Serum samples collected at pre-dose (0 h) and post-dose timepoints (1, 24, 72, 120, 168, 240, 336, and 504 h) were analyzed via ELISA. 96-well plates coated with Goat Anti-Human IgG (0.1 µg/well, Jackson) underwent blocking with 2% BSA to minimize nonspecific binding. Serially diluted standards (50–0.78 ng/mL) and samples were incubated, followed by detection with HRP-conjugated F(ab')₂ Goat Anti-Human IgG (Jackson) and TMB substrate. Reactions were terminated with sulfuric acid, and OD_450_ values were measured. Data analysis using Phoenix 8.1 derived pharmacokinetic parameters from concentration-time curves.

### Statistical analysis

At least three biological replicates were performed for all experiments, unless otherwise specified. The log-rank test was used for survival data analysis. For parametric data, the two-tailed Student’s t-test was used for the analysis of two groups. For nonparametric data, the Mann-Whitney U test was utilized. *P*-values less than or equal to 0.05 were considered statistically significant. Statistical analysis was performed with GraphPad Prism 8.0.2 software.

## SUPPLEMENTAL MATERIAL

Fig. S1 aac.00405-25-s0001.docx Determination of the specificity of 5C8 binding to Pseudomonas aeruginosa by enzyme-linked immunosorbent assay.

Fig. S2 aac.00405-25-s0002.docx Interacting surfaces of 5C8-Fab and PcrV residues D125, K129, Y145.

Fig. S3 aac.00405-25-s0003.docx SDS-PAGE analysis of purified recombinant PcrV mutants under reducing conditions.

Fig. S4 aac.00405-25-s0004.docx Comparative efficacy of 5C8 with clinical-stage anti-PcrV antibodies in a murine bloodstream infection model.

Table S1 aac.00405-25-s0005.docx PCR results for the pcrV, exoU, and exoS genes in clinical strains of *Pseudomonas aeruginosa*.

Table S2 aac.00405-25-s0006.docx Determination of the affinities between PcrV and anti-PcrV mAbs by enzyme-linked immunosorbent assay.

Table S3 aac.00405-25-s0007.docx Effect of mutation in PcrV amino acid residues.

Table S4 aac.00405-25-s0008.docx Primers used for PCR-based detection of T3SS genes.

ASM does not own the copyrights to Supplemental Material that may be linked to, or accessed through, an article. The authors have granted ASM a non-exclusive, world-wide license to publish the Supplemental Material files. Please contact the corresponding author directly for reuse.
