# Supplementary material for: Mechanistic blockade of Pseudomonas aeruginosa type III secretion by a monoclonal antibody targeting the pore size-determining domain of PcrV
Source: Antimicrob Agents Chemother. 2025 Aug 18;69(10):e00405-25. doi: 10.1128/aac.00405-25 (PMC12486813; doi:10.1128/aac.00405-25)
Supplement: Fig. S1 — Determination of the specificity of 5C8 binding to Pseudomonas aeruginosa by enzyme-linked immunosorbent assay. [file aac.00405-25-s0001.docx]

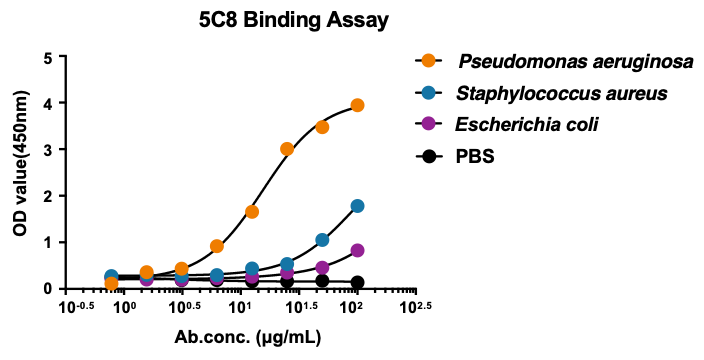


**FIG S1** Determination of the specificity of 5C8 binding to *Pseudomonas aeruginosa* by enzyme-linked immunosorbent assay (ELISA). Serial dilutions of 5C8 were incubated with *Pseudomonas aeruginosa* strain 103753 (1×10⁸ CFU/well). *Staphylococcus aureus* (USA300) and *Escherichia coli* BL21(DE3) (strains without PcrV) served as negative controls, while PBS was used as the blank.
