# Supplementary material for: Mechanistic blockade of Pseudomonas aeruginosa type III secretion by a monoclonal antibody targeting the pore size-determining domain of PcrV
Source: Antimicrob Agents Chemother. 2025 Aug 18;69(10):e00405-25. doi: 10.1128/aac.00405-25 (PMC12486813; doi:10.1128/aac.00405-25)
Supplement: Fig. S2 — Interacting surfaces of 5C8-Fab and PcrV residues D125, K129, Y145. [file aac.00405-25-s0002.docx]

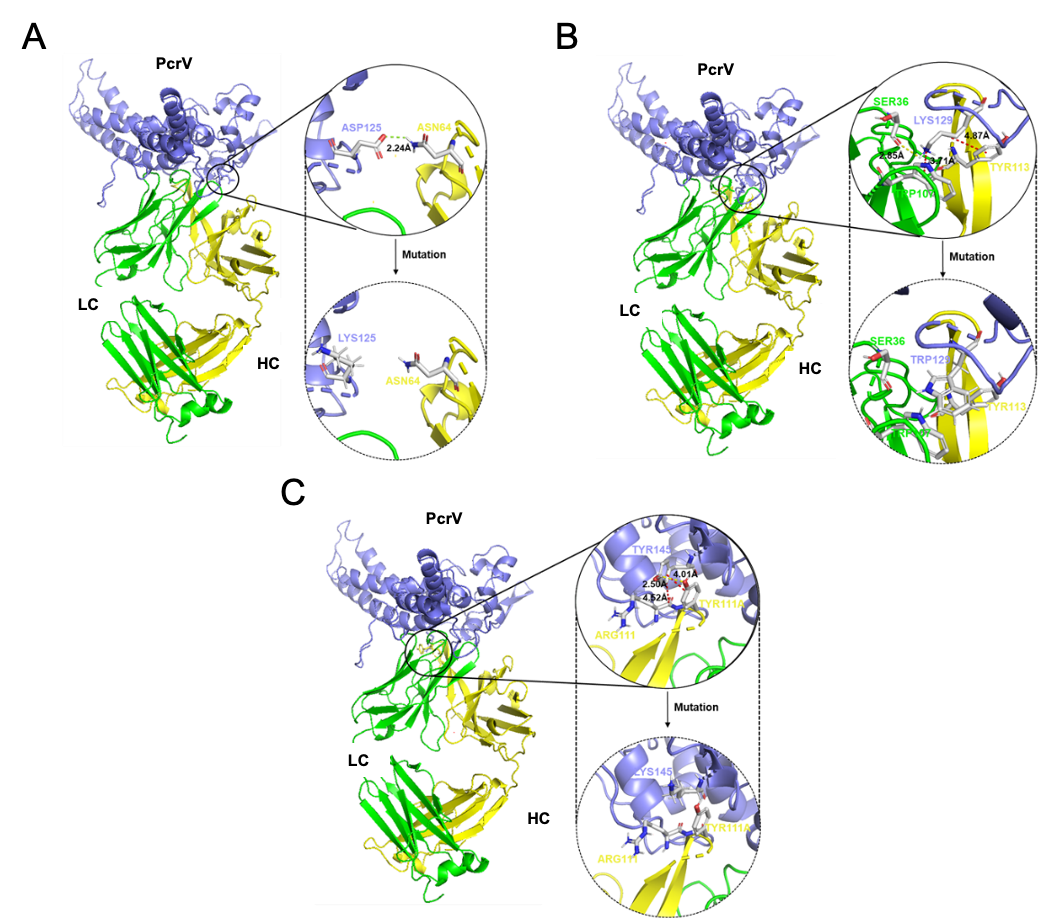


**FIG S2** Interacting surfaces of 5C8-Fab and PcrV residues D125, K129, Y145. (A) Mutation of ASP125 (D125) to LYS125 (K125) results in the loss of the hydrogen bond between PcrV and ASN64 in the heavy chain (HC) of 5C8. (B) When LYS129 (K129) mutates to TRP129 (W129), both the hydrogen bond and π-π stacking interactions between PcrV and SER36 in the light chain (LC), as well as TYR113 in the HC of 5C8, are abolished. (C) Mutation of TYR145 (Y145) to LYS145 (K145) eliminates the hydrogen bond and π-π stacking interactions between PcrV and ARG111 and TYR111A in the HC of 5C8. The LC is green, the HC is yellow, and PcrV is purple. The green dotted line signifies a hydrogen bond, while the red dotted line indicates π-π stacking. ASP, aspartic acid; LYS, lysine; ASN, asparagine; TRP, tryptophan; SER, serine; TYR, tyrosine; ARG, arginine.
