# Supplementary material for: Mechanistic blockade of Pseudomonas aeruginosa type III secretion by a monoclonal antibody targeting the pore size-determining domain of PcrV
Source: Antimicrob Agents Chemother. 2025 Aug 18;69(10):e00405-25. doi: 10.1128/aac.00405-25 (PMC12486813; doi:10.1128/aac.00405-25)
Supplement: Fig. S3 — SDS-PAGE analysis of purified recombinant PcrV mutants under reducing conditions. [file aac.00405-25-s0003.docx]

**
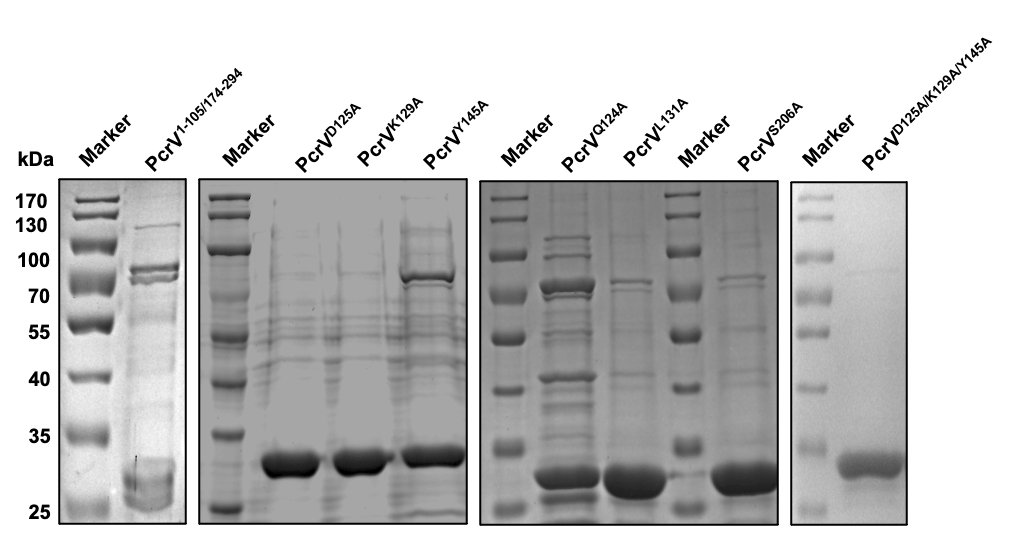
**

**FIG S3.** SDS-PAGE analysis of purified recombinant PcrV mutants under reducing conditions.​​ Coomassie-stained 10% gel showing: deletion mutant (PcrV^1-105/174-294^; theoretical MW ≈ 27 kDa), alanine substitution mutants (PcrV^Q124A^, PcrV^D125A^, PcrV^K129A^, PcrV^L131A^, PcrV^Y145A^, PcrV^S206A^; theoretical MW ≈ 33 kDa), triple mutant (PcrV^D125A/K129A/Y145A^, theoretical MW ≈ 33 kDa). Marker (kDa values as labeled) was included. 10 μg protein loaded per lane. MW, molecular weight.
