# Supplementary material for: Mechanistic blockade of Pseudomonas aeruginosa type III secretion by a monoclonal antibody targeting the pore size-determining domain of PcrV
Source: Antimicrob Agents Chemother. 2025 Aug 18;69(10):e00405-25. doi: 10.1128/aac.00405-25 (PMC12486813; doi:10.1128/aac.00405-25)
Supplement: Fig. S4 — Comparative efficacy of 5C8 with clinical-stage anti-PcrV antibodies in a murine bloodstream infection model. [file aac.00405-25-s0004.docx]

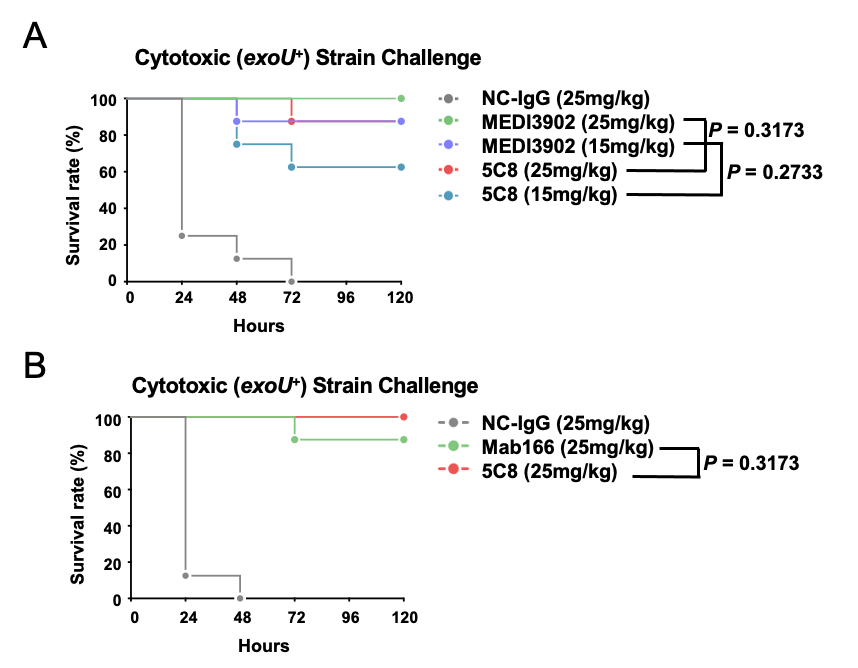


**FIG S4** Comparative efficacy of 5C8 with clinical-stage anti-PcrV antibodies in a murine bloodstream infection model​. (A) Survival curves for mice (n = 8) infected with *Pseudomonas aeruginosa* strain 103753 (7.2×10^7^ CFU/mouse) and treated with 5C8 or MEDI3902 at 25, 15 mg/kg. No statistically significant differences were observed between 5C8 and MEDI3902 by Log-Rank test (25 mg/kg: *P* = .3173; 15 mg/kg: *P* = .273). (B) Head-to-head comparison of 5C8 and the prototype anti-PcrV antibody Mab166 (25 mg/kg) under identical infection conditions. Survival curves showed equivalent protection (*P* = .3173 by Log-Rank test).
