# Supplementary material for: Mechanistic blockade of Pseudomonas aeruginosa type III secretion by a monoclonal antibody targeting the pore size-determining domain of PcrV
Source: Antimicrob Agents Chemother. 2025 Aug 18;69(10):e00405-25. doi: 10.1128/aac.00405-25 (PMC12486813; doi:10.1128/aac.00405-25)
Supplement: Table S1 — PCR results for the pcrV, exoU, and exoS genes in clinical strains of Pseudomonas aeruginosa. [file aac.00405-25-s0005.docx]

**TABLE S1** PCR results for the *pcrV*, *exoU* and *exoS* genes in clinical strains of *Pseudomonas aeruginosa*.

| **Strain ID** | **Clinical Source** | ***exoU* PCR** | ***exoS PCR*** | ***pcrV* PCR** |
| --- | --- | --- | --- | --- |
| 101085 | pus | + | - | + |
| 101553 | sputum | + | - | + |
| 102025 | sputum | + | - | + |
| 102214 | sputum | + | - | + |
| 102411 | sputum | + | - | + |
| 102720 | sputum | + | - | + |
| 102744 | sputum | + | - | + |
| 102830 | sputum | + | - | + |
| 102930 | sputum | + | - | + |
| 103352 | sputum | + | - | + |
| 103490 | sputum | + | - | + |
| 103562 | sputum | + | - | + |
| 103570 | sputum | + | - | + |
| 103653 | bronchoalveolar lavage fluid | + | - | + |
| 103659 | sputum | + | - | + |
| 103753 | sputum | + | - | + |
| 104053 | sputum | + | - | + |
| 104193 | sputum | + | - | + |
| 104375 | sputum | + | - | + |
| 104633 | sputum | + | - | + |
| 104757 | urine | + | - | + |
| 104878 | sputum | + | - | + |
| 105112 | sputum | + | - | + |
| 105197 | sputum | + | - | + |
| 105273 | sputum | + | - | + |
| 105275 | sputum | + | - | + |
| 105276 | sputum | + | - | + |
| 105322 | sputum | + | - | + |
| 105413 | sputum | + | - | + |
| 105741 | sputum | + | - | + |
| 106067 | urine | + | - | + |
| 106203 | sputum | + | - | + |
| 107263 | pus | + | - | + |
| 107979 | sputum | + | - | + |
| 301475 | sputum | + | - | + |
| 301492 | sputum | + | - | + |
| 312686 | sputum | + | - | + |
| 103446 | sputum | + | + | not detected |
| 105775 | bronchoalveolar lavage fluid | + | + | not detected |
| 301477 | sputum | + | + | not detected |
| 100142 | sputum | - | + | not detected |
| 100491 | sputum | - | + | not detected |
| 100586 | sputum | - | + | not detected |
| 100626 | sputum | - | + | not detected |
| 100717 | ascites | - | + | not detected |
| 100902 | sputum | - | + | not detected |
| 100993 | sputum | - | + | not detected |
| 101149 | sputum | - | + | not detected |
| 101150 | sputum | - | + | not detected |
| 101164 | sputum | - | + | not detected |
| 101200 | sputum | - | + | not detected |
| 101221 | sputum | - | + | not detected |
| 101263 | pus | - | + | not detected |
| 101330 | sputum | - | + | not detected |
| 101397 | sputum | - | + | not detected |
| 101412 | sputum | - | + | not detected |
| 101446 | sputum | - | + | not detected |
| 101447 | sputum | - | + | not detected |
| 101579 | sputum | - | + | not detected |
| 101580 | sputum | - | + | not detected |
| 101768 | sputum | - | + | not detected |
| 101824 | sputum | - | + | not detected |
| 101844 | sputum | - | + | not detected |
| 101896 | sputum | - | + | not detected |
| 101943 | sputum | - | + | not detected |
| 101988 | sputum | - | + | not detected |
| 101989 | sputum | - | + | not detected |
| 102065 | sputum | - | + | not detected |
| 102169 | sputum | - | + | not detected |
| 102173 | bile | - | + | not detected |
| 102174 | pus | - | + | not detected |
| 102429 | sputum | - | + | not detected |
| 102503 | sputum | - | + | not detected |
| 102574 | sputum | - | + | not detected |
| 102653 | pus | - | + | not detected |
| 102654 | bile | - | + | not detected |
| 102699 | sputum | - | + | not detected |
| 103108 | sputum | - | + | not detected |
| 103113 | sputum | - | + | not detected |
| 103454 | pus | - | + | not detected |
| 103569 | sputum | - | + | not detected |
| 103700 | pus | - | + | not detected |
| 103871 | pus | - | + | not detected |
| 103887 | sputum | - | + | not detected |
| 104074 | urine | - | + | not detected |
| 104116 | sputum | - | + | not detected |
| 104180 | pus | - | + | not detected |
| 104341 | sputum | - | + | not detected |
| 104529 | sputum | - | + | not detected |
| 104934 | sputum | - | + | not detected |
| 105024 | sputum | - | + | not detected |
| 105197 | sputum | - | + | not detected |
| 105473 | sputum | - | + | not detected |
| 105550 | sputum | - | + | not detected |
| 105574 | sputum | - | + | not detected |
| 105624 | sputum | - | + | not detected |
| 105636 | sputum | - | + | not detected |
| 105685 | sputum | - | + | not detected |
| 105871 | sputum | - | + | not detected |
| 105931 | sputum | - | + | not detected |
| 105943 | pus | - | + | not detected |
| 106014 | sputum | - | + | not detected |
| 106032 | sputum | - | + | not detected |
| 106058 | pus | - | + | not detected |
| 106278 | sputum | - | + | not detected |
| 106333 | pus | - | + | not detected |
| 106338 | wound | - | + | not detected |
| 106358 | pus | - | + | not detected |
| 106467 | urine | - | + | not detected |
| 106527 | sputum | - | + | not detected |
| 106583 | sputum | - | + | not detected |
| 106621 | sputum | - | + | not detected |
| 106992 | sputum | - | + | not detected |
| 107085 | urine | - | + | not detected |
| 107113 | sputum | - | + | not detected |
| 107121 | sputum | - | + | not detected |
| 107231 | sputum | - | + | not detected |
| 107246 | sputum | - | + | not detected |
| 301476 | sputum | - | + | not detected |
| 305711 | sputum | - | + | not detected |
| 306800 | sputum | - | + | not detected |
| 306899 | sputum | - | + | not detected |
| 306902 | sputum | - | + | not detected |
| 100171 | sputum | - | - | not detected |
| 100483 | sputum | - | - | not detected |
| 100756 | sputum | - | - | not detected |
| 100858 | sputum | - | - | not detected |
| 100889 | sputum | - | - | not detected |
| 100997 | sputum | - | - | not detected |
| 101341 | sputum | - | - | not detected |
| 101365 | sputum | - | - | not detected |
| 101366 | sputum | - | - | not detected |
| 101894 | pus | - | - | not detected |
| 102654 | bile | - | - | not detected |
| 103096 | catheter | - | - | not detected |
| 103152 | sputum | - | - | not detected |
| 103452 | pus | - | - | not detected |
| 104119 | sputum | - | - | not detected |
| 104166 | sputum | - | - | not detected |
| 104405 | sputum | - | - | not detected |
| 104634 | sputum | - | - | not detected |
| 105177 | sputum | - | - | not detected |
| 105274 | sputum | - | - | not detected |
| 105591 | sputum | - | - | not detected |
| 105694 | sputum | - | - | not detected |
| 106637 | sputum | - | - | not detected |
| 304594 | sputum | - | - | not detected |
