# Supplementary material for: Mechanistic blockade of Pseudomonas aeruginosa type III secretion by a monoclonal antibody targeting the pore size-determining domain of PcrV
Source: Antimicrob Agents Chemother. 2025 Aug 18;69(10):e00405-25. doi: 10.1128/aac.00405-25 (PMC12486813; doi:10.1128/aac.00405-25)
Supplement: Table S2 — Determination of the affinities between PcrV and anti-PcrV mAbs by enzyme-linked immunosorbent assay. [file aac.00405-25-s0006.docx]

**TABLE S2** Determination of the affinities between PcrV and anti-PcrV mAbs by enzyme-linked immunosorbent assay (ELISA).

| **Antigen** | **mAb** | **EC_50_ (ng/mL) ^*^** |
| --- | --- | --- |
| PcrV | 16E5 | 7.414 |
|  | 5C8 | 7.787 |
|  | 21D6 | 11.12 |
|  | 7D9 | 5.732 |
|  | 1A11 | 39.56 |
|  | 1B4 | 7.231 |
|  | 22H10 | 9.507 |
|  | 32A7 | 11.84 |

*The EC_50_ value represents the average of two independent experiments. Abbreviations: EC_50_, median effective concentration; mAb: monoclonal antibody.
