# Supplementary material for: Mechanistic blockade of Pseudomonas aeruginosa type III secretion by a monoclonal antibody targeting the pore size-determining domain of PcrV
Source: Antimicrob Agents Chemother. 2025 Aug 18;69(10):e00405-25. doi: 10.1128/aac.00405-25 (PMC12486813; doi:10.1128/aac.00405-25)
Supplement: Table S3 — Effect of mutation in PcrV amino acid residues. [file aac.00405-25-s0007.docx]

**TABLE S3** Effect of mutation in PcrV amino acid residues.

| **Mutation** | **Mutation Energy** | **Effect of Mutation *** |
| --- | --- | --- |
| :GLN124>ALA | -1.10 | STABILIZING |
| :ASP125>ALA | 0.53 | DESTABILIZING |
| :LYS129>ALA | 1.14 | DESTABILIZING |
| :TYR145>ALA | 1.07 | DESTABILIZING |
| :SER206>ALA | -0.20 | NEUTRAL |
| :LEU131>ALA | 0.01 | NEUTRAL |

*The Effect of Mutation can be classified as: DESTABILIZING (when Mutation Energy exceeds 0.5), resulting in a decrease in affinity; NEUTRAL (when Mutation Energy ranges from -0.5 to +0.5), indicating no influence on affinity; and STABILIZING (when Mutation Energy is below -0.5), leading to an increase in affinity. Abbreviations: GLN, glutamine; ALA, alanine; ASP, aspartic acid; LYS, lysine; TYR, tyrosine; SER, serine; LEU, leucine.
