# Supplementary material for: Mechanistic blockade of Pseudomonas aeruginosa type III secretion by a monoclonal antibody targeting the pore size-determining domain of PcrV
Source: Antimicrob Agents Chemother. 2025 Aug 18;69(10):e00405-25. doi: 10.1128/aac.00405-25 (PMC12486813; doi:10.1128/aac.00405-25)
Supplement: Table S4 — Primers used for PCR-based detection of T3SS genes. [file aac.00405-25-s0008.docx]

**TABLE S4.** Primers used for PCR-based detection of T3SS genes.

| **Gene** | **Primers for amplification assays** | **Length** | **Primers for detection assays** | **Length** |
| --- | --- | --- | --- | --- |
| ***pcrV*** | F: 5’-CGTGGCTTGTTGATCTGAGG-3’ | 935 bp | F: 5’-GTTCCTGGTGTCGGCCTATT-3’ | 325 bp |
|  | R: 5’-GGTCGGCTGGTTCATGGATAC-3’ |  | R: 5’-CTTGCCGCTGAAGGTATCCA-3’ |  |
| ***exoU*** | F: 5’-GCTCCGAACCCTCGGTATTT-3’ | 2192 bp | F: 5’-TGGCGCTAGAAGAGAAAGGC-3’ | 459 bp |
|  | R: 5’-CCATAACCAGTTCCGGTCCC-3’ |  | R: 5’-TGGCCGTACCTGTGATGTTC-3’ |  |
| ***exoS*** | F: 5’-TGGCGGAGAAACATCAGGAG-3’ | 1447 bp | F: 5’-AGAGCGAGGTCAGCAGAGTA-3’ | 355 bp |
|  | R: 5’-AAGAATCGGACACCCCAAGG-3’ |  | R: 5’-GAGAGATAGCCGTCGTCGTG-3’ |  |
